# Association of NK cell subsets and cytotoxicity with *FCGR3A* gene polymorphism in functional NK cell deficiency

**DOI:** 10.1590/1806-9282.20230872

**Published:** 2024-02-26

**Authors:** Mehmet Ali Karaselek, Ercan Kurar, Sevgi Keleş, Şükrü Nail Güner, İsmail Reisli

**Affiliations:** 1 Necmettin Erbakan University, Faculty of Medicine, Department of Medical Biology - Konya, Turkey.; 2 Necmettin Erbakan University, Faculty of Medicine, Department of Pediatric Immunology and Allergy - Konya, Turkey.

**Keywords:** FCGR3A, NK, Polymorphism, Cytotoxicity

## Abstract

**OBJECTIVE:**

The purpose of this study was to assess the association between clinical, laboratory, and functional analyses and polymorphism in the *FCGR3A* gene in individuals with functional NK cell deficiency.

**METHODS:**

A total of 15 functional NK cell deficiency patients and 10 age-matched healthy controls underwent NK cell subgroup, cytotoxicity, and *FCGR3A* whole-exome analysis with next-generation sequencing.

**RESULTS:**

Three different NK cell subsets (CD56^bright^CD16^neg^, CD56^bright^CD16^int^, and CD56^dim^CD16^hi^) were identified. No statistically significant difference was found in the ratio of CD56^bright^CD16^neg^ cells between patients and controls. CD56^bright^CD16^int^ and CD56^dim^CD16^hi^ ratios were found to be significantly lower in patients. As a result of NK cell cytotoxicity analysis, a proportional decrease of K562 amount between patients and controls was found to be statistically significant (p<0.001). In the *FCGR3A* whole-exome analysis, all patients were found to be homozygous mutant for the c.526G > T (p.V176F) in exon 4, while three patients were homozygous wild type and 12 patients were heterozygous for the c.197T>A (p.L66H) in exon 3.

**CONCLUSION:**

In this study, a group of pediatric patients with suspected functional NK cell deficiency were evaluated and the findings indicated that NK subsets, cytotoxicity results, and *FCGR3A* gene polymorphism were found to be correlated with the clinical features. We conclude that this kind of study might contribute to follow-up the patients in time.

## INTRODUCTION

Mature NK cells (CD3-CD56^+^) defend against viral infections and tumors^1^ ^,^ ^2^ ^,^ ^3^. Initially, peripheral blood (PB) NK cells were classified as CD56^bright^ and CD56^dim^ ^4^. Further studies refined this classification into CD56^bright^CD16^neg^, CD56^bright^CD16^int^, and CD56^dim^CD16^hi^ subsets^5^ ^,^ ^6^ ^,^ ^7^ ^,^ ^8^ ^,^ ^9^.

The *FCGR3A* (NM_000569) gene on chromosome 1 has five exons, is of 8345 bp size, and encodes CD16, a low-affinity receptor (50-70 kDa) that binds to the Fc region of IgG. *FCGR3A* is expressed in macrophages, γδ T cells, and mainly NK cells^6^. CD16 plays a role in antibody-dependent cellular cytotoxicity (ADCC) in NK cells^10^. The homozygous p.T230A substitution has no impact on CD16 expression but hinders detection with B73.1 monoclonal antibody (mAb), impairing NK cell cytotoxicity. This substitution is linked to functional NK cell deficiency (FNKD)^11^ ^,^ ^12^. FNKD is characterized by non-functional NK despite the normal range of mature NK cells in the PB and was first described in two patients with recurrent upper respiratory tract and herpes simplex virus (HSV) infections. Genetic and functional analysis of the patients showed that the ADCC function of NK cells was not impaired, their cytotoxicity was impaired, and p.L66H missense mutation was detected in the FcγRIIIA gene^2^ ^,^ ^13^. Although there is sensitivity to viral infections in FNKD, the most common finding is recurrent upper respiratory tract infection^12^. Although the population of CD56^dim^ and CD56^bright^ cells, which are NK cell subsets, is reported to be variable, there is still no clear data^14^.

A common polymorphism at position 176 (p.V176F) influences CD16’s IgG Fc affinity^12^. From this point of view, it was aimed to investigate the relationship between NK cell subsets and NK cell cytotoxicity and *FCGR3A* polymorphism in FNKD.

## METHODS

### Patients

The study included FNKD patients (n:15) admitted to our clinic between 2016 and 2018, who had viral infections (influenza, rhinovirus, respiratory syncytial virus A-B, herpes virus, and metapneumovirus), undetectable CD16 expression by B73.1 mAb on NK cells (despite normal CD16 expression by 3G8 mAb), and age-matched healthy control (n:10).

### NK cell subset analysis

Peripheral blood mononuclear cells (PBMCs) were isolated from patients and controls using Ficoll-Hypaque with gradient centrifugation (Sigma-Aldrich, Steinheim, Germany). Surface staining utilized anti-CD3 (V500C), anti-CD16 (PE), and anti-CD56 (APC) mAbs. CD16 expressions of patients and controls were evaluated with mAbs from two different clones (i.e., B73.1 and 3G8). Flow cytometry analysis was performed using a BD Biosciences Canto II device (at least 10´10^3^ cells from each subject) with the FACSDiva software version 6.1.3. The analysis revealed three NK cell subsets: CD56^bright^CD16^neg^, CD56^bright^CD16^int^, and CD56^dim^CD16^hi^.

### NK cell cytotoxicity analysis

NK cell cytotoxicity was assessed using the NKTEST^®^ kit (Catalog number: 15991230, Glycotope Biotechnology, Heidelberg, Germany) with varying E:T ratios [NK as effector cells, (E); K562 as target cells, (T)] and analyzed by flow cytometry (BD Biosciences, Heidelberg, Germany) using the FACSDiva software version 6.1.3.

### *FCGR3A* whole-exome analysis

Whole-gene sequencing of the *FCGR3A* gene was performed using next-generation sequencing (NGS) technology. Bioinformatic analysis revealed approximately 98% ortholog between *FCGR3A* (NM_000569) and *FCGR3B* (NM_000570) genes. To prevent potential amplification of *FCGR3B*, two separate primer pairs were designed for exons 1-3 and exons 4-5 (primers for exons 1-3; F: AAATCACACTAAAAAGTCAGTAGCTCC, R: ACTTTGGGAAGCCAAGGCTG; primers for exons 4-5; F: CCATGCTCAGTAAATTACTTGGTG, R: ATTTAGGAATAATTGTTTTTTTTTCCC). Polymorphisms detected in two different regions of the *FCGR3A* gene were validated by Sanger sequencing (for c.526G > T (p.V176F); F: ACTTTTGGGGACCTCCTGGT, R: TCACAGCTGGAAGAACACTGC; for c.197T>A (p.L66H); F: TGGGACCACACATCATCTCA, R: CAAAGGCTGTGGTGTTCCTG).

**Figure 1.**
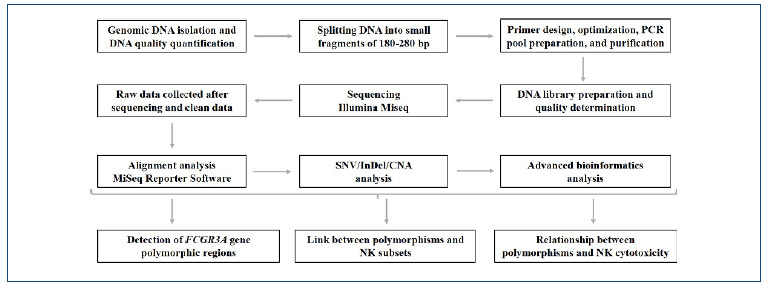
Presentation of the detailed data interpretation and analysis.

After primer design, optimization was performed, and a PCR pool was created. Purification of the PCR pool was done using the NucleoFast^®^ 96 PCR kit (Cat. no. 740786, Macherey-Nagel GmbH, Germany). The resulting DNA was quantified (Nanodrop ND-1000, Thermo Inc.) and standardized to 0.5 ng/µL. DNA library preparation utilized the NexteraXT DNA Library Prep Kit (Cat. no. FC-131-1024, Macherey-Nagel Gmbh, Germany). Illumina Miseq NGS (Illumina, San Diego, CA, USA) was used for sequencing, and data analysis was performed with the MiSeq Reporter Software (Illumina Inc.) and IGV 2.5.0 software (Broad Institute) using the hg19 human reference genome (Figure 1)^15^ ^,^ ^16^.

### Statistical analysis

Normality assumption was assessed using the Shapiro-Wilk test. Two-way repeated-measures ANOVA and Mann-Whitney U test compared concentration changes in patient and control groups in NK cell cytotoxicity. Mauchly’s test of sphericity evaluated the sphericity assumption. The within-subjects effects table assessed concentration-dependent changes, main effects, and interactions with groups. Simple effect tests compared concentrations within each group. Bonferroni correction controlled multiple comparisons. Results included F-test values and partial eta square (η^2^) for effect size. Pearson correlation examined the relationship between NK cell subgroups and cytotoxicity. p<0.05 indicated statistical significance. Analyses were performed by the JASP Team (Version 0.11.1; 2019) software.

## RESULTS

A total of 15 patients (11 males and 4 females) and 10 age-matched controls (5 males and 5 females) were included. There was no significant difference in age and gender (p>0.05). The admission age was a median of 9.5 months (2-144±42), and the study age was a median of 2 years (2-15±5). Seven patients presented with frequent illness, five patients with recurrent bronchiolitis, two patients with recurrent resistant fever, and one patient with persistent wounds on the face and recurrent fever. When the clinical histories of the patients are evaluated, it is noteworthy that the most common findings are fever, pneumonitis, and bronchiolitis. Eleven patients had a history of hospitalization due to infection. Six patients had consanguineous marriages, and eight patients had a family history of death from an unknown cause in infancy. In addition, as recurrent infections observed in patients may be related to dysfunction of anatomical and physiological barriers and allergic diseases, these findings were also evaluated and these conditions were excluded. Other immunological tests (e.g., immunoglobulin levels, lymphocyte subsets, vaccine responses, isohemagglutinin titers) were normal.

### NK and NK cell subgroup analysis

Patients had significantly lower total NK cell rates (6.7%) compared with controls (18.8%, p=0.002). CD16 expressions were also significantly lower in patients (p<0.001).

**Figure 2.**
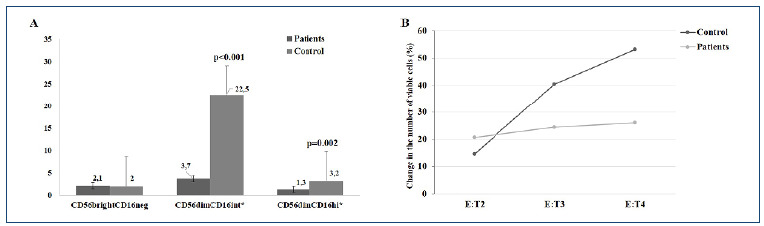
(A) Distribution of NK subgroups in patients and controls. (B) Variation of different ratios in target cell amount in NK cell cytotoxicity analysis (*statistically significant; E:T: effector: target cell ratio).

CD56^bright^CD16^neg^ ratios did not differ significantly between patients and controls (p=0.931). CD56^bright^CD16^int^ (p<0.001) and CD56^dim^CD16^hi^ (p=0.002) ratios were significantly lower in patients (Figure 2A).

### NK cell cytotoxicity

In the control group, the decrease in target cell ratio was 3.07-fold between E:T4 (13%) and E:T3 (40%) and 4.07-fold between E:T4 and E:T2 (53%). In the patient group, the average decrease was 1.25-fold between these rates. These results suggest reduced NK cell cytotoxicity in the patient (Figure 2B).

Correlation analysis was performed for E:T2 and E:T3 concentrations in NK cell cytotoxicity. A stronger correlation was observed in E:T2 for overall cytotoxicity. Positive correlations were found between CD56b^right^CD16^int^ and CD56^dim^CD16^hi^ subsets and NK cell cytotoxicity (p=0.044 and p=0.018). A non-significant negative correlation was found between CD56^bright^CD16^neg^ subset and cytotoxicity (p=0.433) (Table 1).

**Table 1.** Pearson correlation analysis results.

NK cell subsets	Effector and target cell ratios	
	E:T3	E:T2
CD56^bright^CD16^neg^	p: 0.233	p: 0.433
	r: -0.306	r: -0.191
CD56^bright^CD16^int^	p: 0.546	p: 0.044*
	r: 0.158	r: 0.493
CD56^dim^CD16^hi^	p: 0.113	p: 0.018*
	r: 0.399	r: 0.546

E:T: effector and target ratio, *p<0.05.

### *FCGR3A* whole-gene analysis

NGS analysis of the *FCGR3A* gene showed homozygosity for p.V176F (rs396991; g.16151454 A>C; p.V176F) in exon 4 in all FNKD patients and heterozygosity in controls. In exon 3 (rs10127939; g.161518333 A>C, A>T; p.L66H), different genotypes (homozygous wild type n: 3 and heterozygous n: 12) were observed in patients.

### Correlation analysis of the results

Correlation analysis revealed a negative correlation between CD56^bright^CD16^neg^ NK subset and NK cell cytotoxicity (E:T3: r=-0.306, p=0.233; E:T2: r=-0.191, p=0.463). Conversely, a positive correlation was found between CD56^bright^CD16^int^ and CD56^dim^CD16^hi^ NK subsets and NK cell cytotoxicity (r=0.493, p=0.044 and r=0.546, p=0.018, respectively).

CD56^bright^CD16^neg^ rates were similar between patients with homozygous wild-type genotype for exon 3 (heterozygous: 1.88%, homozygous wild type: 1.25%). However, rates differed in CD56^bright^CD16^int^ and CD56^dim^CD16^hi^ subgroups (CD56^bright^CD16^int^: heterozygous genotype: 2.71%, homozygous wild genotype: 8.9%; CD56^dim^CD16^hi^: heterozygous genotype: 1.28%, homozygous wild genotype: 3.15%). NK cell cytotoxicity analysis revealed insufficient cytotoxicity in patients with both genotypes compared with the control.

## DISCUSSION

FNKD patients were assessed for NK cell subsets, cytotoxicity, and *FCGR3A* polymorphism. The study aimed to investigate low cytotoxicity in patients compared with controls, focusing on *FCGR3A* polymorphism. Correlations were found among NK subgroups, cytotoxicity, gene polymorphism, and clinical features.

The literature suggests variations in NK cell subsets with gene polymorphism in NKD^10^ ^,^ ^17^. CD16 expression differences in cases with these polymorphisms are not well studied. Our study detected CD16 epitope loss and consistent NK cell subset results. NK cell rates were similar between patients and controls (p=0.002) and within the normal range for age, aligning with the literature^18^ ^,^ ^19^.

NK cell subsets analysis revealed no significant difference in CD56^bright^CD16^neg^ between groups, but significant differences were observed in CD56^bright^CD16^int^ and CD56^dim^CD16^hi^ subsets. Limited literature is available on the normal ranges of NK cell subsets. Angelo et al., reported CD56^bright^CD16^neg^ as 6.9-8.56% in 40 healthy individuals^5^. In our study, the control group had a lower rate of CD56^bright^CD16^neg^ (2.04%) compared with the literature. Notably, our study involved a pediatric group, which may contribute to the variation in CD56^bright^CD16^neg^ levels compared with Angelo et al.’s study conducted with adult donors^5^.

CD56^bright^ cells are precursors of CD56^dim^ NK cells^6^ ^,^ ^20^ ^,^ ^21^ and exhibit high proliferative capacity^21^ ^,^ ^22^ ^,^ ^23^. The lack of significant difference in CD56^bright^CD16^neg^ between patients and controls suggests normal development of NK cells up to the CD56^bright^ stage. CD56^bright^ NK cells have potent cytokine secretion, while CD56^dim^ subsets are responsible for natural cytotoxicity^18^ ^,^ ^19^. CD56^dim^CD16^dim^ cells were more degranulated than CD56^dim^CD16^bright^ cells in PB. In patients, low CD56^dim^CD16^hi^ cells were consistent with the previous literature^22^ ^,^ ^24^.

The literature suggests that the CD56^dim^ subset is responsible for NK cell cytotoxicity^18^ ^,^ ^19^. However, this is the first study to examine this in the pediatric group, and there are no data on the effectiveness of different gating methods. Correlation analysis showed a negative correlation between CD56^bright^CD16^neg^ NK subset and cytotoxicity and a positive correlation between CD56^bright^CD16^int^ and CD56^dim^CD16^hi^. Results align with patient cell counts. CD56^bright^CD16^int^ counts (patients: 3.7% and controls: 22.5%) and CD56^dim^CD16^hi^ counts (patients: 1.3% and controls: 3.2%) differed significantly (p<0.001), indicating their role in NK cell cytotoxicity.

CD16’s role in NK cell cytotoxicity was demonstrated. CD56^dim^CD16^neg^ expression negatively affects cytotoxicity, while CD16-expressing cells have a positive impact. CD56^bright^CD16^neg^ subgroup negatively affects cytotoxicity. Correlation analysis implies that NK cell cytotoxicity can be assessed without specific analysis.

*FCGR3A* gene sequencing revealed exon 3 variations. Heterozygous genotype was found in 12 patients, while three patients had homozygous normal genotype. Clone B73.1 did not detect CD16 expression in any of these patients, implying that additional unidentified polymorphism may cause epitope loss^11^ ^,^ ^25^. Epitope loss with B73.1 mAb is not solely caused by the p.L66H polymorphism, and other polymorphism/mutations may also contribute. Findings align with Lenart et al.’s study, indicating the presence of additional gene mutations causing CD16 epitope loss^25^. Polymorphic changes were observed in exon 4. Transversion in the *FCGR3A* gene led to the increased binding affinity of NK cells to IgG1 or IgG3 antibodies, affecting NK cell-mediated ADCC. Extreme polymorphism in this region has been observed in different populations but lacks data on the Turkish population. Patients in the study exhibited homozygous wild-type or heterozygous genotypes for exon 3 and mutant homozygous genotype for exon 4.

## CONCLUSION

This study assessed patients with suspected FNKD using comprehensive functional and genetic analyses. NK cell cytotoxicity analysis, despite its complexity, plays a crucial role in FNKD diagnosis. Correlating NK cell subsets with cytotoxicity results can aid in predicting NK cell cytotoxicity. *FCGR3A* gene sequencing involved a limited number of patients and controls, but detecting mutations is essential for disease diagnosis and patient monitoring.

## References

[1] Miller JS (2001). The biology of natural killer cells in cancer, infection, and pregnancy. Exp Hematol.

[2] Orange JS, Ballas ZK (2006). Natural killer cells in human health and disease. Clin Immunol.

[3] Björkström NK, Strunz B, Ljunggren HG (2022). Natural killer cells in antiviral immunity. Nat Rev Immunol.

[4] Lanier LL, Le AM, Phillips JH, Warner NL, Babcock GF (1983). Subpopulations of human natural killer cells defined by expression of the Leu-7 (HNK-1) and Leu-11 (NK-15) antigens. J Immunol.

[5] Angelo LS, Banerjee PP, Monaco-Shawver L, Rosen JB, Makedonas G, Forbes LR (2015). Practical NK cell phenotyping and variability in healthy adults. Immunol Res.

[6] Cooper MA, Fehniger TA, Caligiuri MA (2001). The biology of human natural killer-cell subsets. Trends Immunol.

[7] Michel T, Poli A, Cuapio A, Briquemont B, Iserentant G, Ollert M (2016). Human CD56bright NK cells: an update. J Immunol.

[8] Cosan F, Aktas Cetin E, Akdeniz N, Emrence Z, Cefle A, Deniz G (2017). Natural killer cell subsets and their functional activity in Behçet’s disease. Immunol Invest.

[9] Dobbs K, Tabellini G, Calzoni E, Patrizi O, Martinez P, Giliani SC (2017). Natural killer cells from patients with recombinase-activating gene and non-homologous end joining gene defects comprise a higher frequency of CD56bright NKG2A+++ Cells, and yet display increased degranulation and higher perforin content. Front Immunol.

[10] Mace EM, Orange JS (2016). Genetic causes of human NK cell deficiency and their effect on NK cell subsets. Front Immunol.

[11] Jawahar S, Moody C, Chan M, Finberg R, Geha R, Chatila T (1996). Natural Killer (NK) cell deficiency associated with an epitope-deficient Fc receptor type IIIA (CD16-II). Clin Exp Immunol.

[12] Grier JT, Forbes LR, Monaco-Shawver L, Oshinsky J, Atkinson TP, Moody C (2012). Human immunodeficiency-causing mutation defines CD16 in spontaneous NK cell cytotoxicity. J Clin Invest.

[13] Orange JS (2013). Natural killer cell deficiency. J Allergy Clin Immunol.

[14] Vries E, Koene HR, Vossen JM, Gratama JW, Borne AE, Waaijer JL (1996). Identification of an unusual Fc gamma receptor IIIa (CD16) on natural killer cells in a patient with recurrent infections. Blood.

[15] Dai Y, Liang S, Dong X, Zhao Y, Ren H, Guan Y (2019). Whole exome sequencing identified a novel DAG1 mutation in a patient with rare, mild and late age of onset muscular dystrophy-dystroglycanopathy. J Cell Mol Med.

[16] Zhang R, Chen S, Han P, Chen F, Kuang S, Meng Z (2020). Whole exome sequencing identified a homozygous novel variant in CEP290 gene causes Meckel syndrome. J Cell Mol Med.

[17] Mahapatra S, Mace EM, Minard CG, Forbes LR, Vargas-Hernandez A, Duryea TK (2017). High-resolution phenotyping identifies NK cell subsets that distinguish healthy children from adults. PLoS One.

[18] Maria A, Bozzano F, Cantoni C, Moretta L (2011). Revisiting human natural killer cell subset function revealed cytolytic CD56(dim)CD16+ NK cells as rapid producers of abundant IFN-gamma on activation. Proc Natl Acad Sci U S A.

[19] Nielsen N, Ødum N, Ursø B, Lanier LL, Spee P (2012). Cytotoxicity of CD56(bright) NK cells towards autologous activated CD4+ T cells is mediated through NKG2D, LFA-1 and TRAIL and dampened via CD94/NKG2A. PLoS One.

[20] Bryceson YT, Fauriat C, Nunes JM, Wood SM, Björkström NK, Long EO (2010). Functional analysis of human NK cells by flow cytometry. Methods Mol Biol.

[21] Brenchley JM, Karandikar NJ, Betts MR, Ambrozak DR, Hill BJ, Crotty LE (2003). Expression of CD57 defines replicative senescence and antigen-induced apoptotic death of CD8+ T cells. Blood.

[22] Matos ME, Schnier GS, Beecher MS, Ashman LK, William DE, Caligiuri MA (1993). Expression of a functional c-kit receptor on a subset of natural killer cells. J Exp Med.

[23] Romagnani C, Juelke K, Falco M, Morandi B, D’Agostino A, Costa R (2007). CD56brightCD16- killer Ig-like receptor- NK cells display longer telomeres and acquire features of CD56dim NK cells upon activation. J Immunol.

[24] Amand M, Iserentant G, Poli A, Sleiman M, Fievez V, Sanchez IP (2017). Human CD56dimCD16dim cells as an individualized natural killer cell subset. Front Immunol.

[25] Lenart M, Trzyna E, Rutkowska M, Bukowska-Strakova K, Szaflarska A, Pituch-Noworolska A (2010). The loss of the CD16 B73.1/Leu11c epitope occurring in some primary immunodeficiency diseases is not associated with the FcgammaRIIIa-48L/R/H polymorphism. Int J Mol Med.

